# Evaluating the Accuracy of Point-of-Care Ultrasound for Peripheral Intravenous Cannulation in Emergency and Trauma Patients: A Systematic Review

**DOI:** 10.7759/cureus.83625

**Published:** 2025-05-07

**Authors:** Shimaa T Elshikh, Binu Thomas, Mawada Taha, Abdul Mueed Shaikh, Aliaa H Alkhazendar, Manahil Awan, Shahzad Ahmad, Jarallah H.J. AlKhazendar

**Affiliations:** 1 Medicine, University of Bahri, Khartoum, SDN; 2 Urology, KIMS Alshifa Super Speciality Hospital, Perintalmanna, IND; 3 General Surgery, The National Ribat University, Khartoum, SDN; 4 Orthopedics, Liaquat National Hospital, Karachi, PAK; 5 Surgery, The Islamic University of Gaza, Gaza, PSE; 6 Executive and Special Ward, Liaquat National Hospital, Karachi, PAK; 7 Surgery, Liaquat National Hospital, Karachi, PAK; 8 General and Emergency Surgery, East and North Hertfordshire NHS Trust, Lister Hospital, Stevenage, GBR

**Keywords:** diva, emergency medicine, nurse training, peripheral intravenous access, pocus, point-of-care ultrasound, trauma, ultrasound-guided cannulation, vascular access

## Abstract

This systematic review evaluates the accuracy and effectiveness of point-of-care ultrasound (POCUS) for peripheral intravenous (PIV) line placement in trauma and emergency department patients. PIV access can be challenging in patients with difficult vascular access (DIVA), often leading to delays in care. POCUS has emerged as a promising modality to improve success rates and reduce complications in such scenarios. A comprehensive literature search was conducted in accordance with the Preferred Reporting Items for Systematic Reviews and Meta-Analyses (PRISMA) guidelines across PubMed, EMBASE, Scopus, and Google Scholar. Five studies met the inclusion criteria, encompassing randomized controlled trials, observational studies, a quality improvement initiative, and a meta-analysis, with a combined total of over 2,800 patients. The findings consistently demonstrated that POCUS significantly improves first-attempt success rates, decreases the number of cannulation attempts, and enhances procedural efficiency. One randomized trial reported a faster median cannulation time with bi-plane imaging (35 seconds vs. 45 seconds, p = 0.03), while a meta-analysis showed a two-fold increase in the odds of first-pass success using ultrasound guidance (OR: 2.1; 95% CI: 1.65-2.7; p < 0.001). Nurse-led POCUS training programs also proved highly effective, achieving post-training success rates above 90%. Despite some limitations, such as heterogeneity in study design, small sample sizes, and mixed pediatric and adult populations, the overall evidence supports the integration of POCUS into emergency protocols for vascular access. The results suggest that routine POCUS use can improve outcomes and workflow, particularly in time-sensitive trauma care.

## Introduction and background

Point-of-care ultrasound (POCUS) has become an invaluable tool in emergency and critical care settings, allowing for real-time, bedside imaging that aids in both diagnostic and procedural interventions. Among its many applications, POCUS has gained prominence in facilitating peripheral intravenous (PIV) access, especially in patients with difficult vascular anatomy or urgent resuscitation needs [1]. Trauma patients, often presenting with hypovolemia, vascular collapse, or altered consciousness, frequently pose challenges for rapid intravenous access. In such scenarios, traditional landmark-based techniques may result in multiple failed attempts, procedural delays, and increased risk of complications. The use of POCUS for guiding PIV placement has the potential to improve first-attempt success rates, reduce time to access, and minimize associated complications, making it a valuable technique in trauma resuscitation protocols.

Existing literature has demonstrated the benefits of ultrasound-guided PIV access in various patient populations, including pediatric, septic, and critically ill cohorts. However, trauma patients present unique anatomical and physiological challenges that may influence the utility and effectiveness of POCUS [2]. Furthermore, there remains variability in training protocols, operator experience, and technique standardization across emergency departments. While some randomized and observational studies support the superiority of ultrasound-guided techniques over traditional methods, the evidence specific to trauma settings remains heterogeneous and fragmented. A focused synthesis of existing studies is necessary to better understand the diagnostic accuracy, procedural success, and overall outcomes of POCUS-guided PIV access in trauma populations.

To address this gap, we conducted a systematic review to evaluate the accuracy and effectiveness of POCUS in facilitating PIV line placement, specifically in trauma patients. The PICO (Population, Intervention, Comparison, and Outcome) framework [3] guiding this review is as follows: P - trauma patients requiring peripheral IV access; I - use of POCUS guidance; C - conventional landmark-based IV placement; O - accuracy, success rate, first-attempt success, and procedural outcomes.

## Review

Materials and methods

Search Strategy

This systematic review was conducted in accordance with the Preferred Reporting Items for Systematic Reviews and Meta-Analyses (PRISMA) guidelines [4] to ensure methodological transparency and reproducibility. A comprehensive literature search was performed across multiple electronic databases, including PubMed, EMBASE, Scopus, and Google Scholar, covering studies published up to August 2024. Keywords and MeSH terms used in various combinations included “point-of-care ultrasound”, “POCUS”, “peripheral intravenous”, “vascular access”, “emergency department”, and “trauma”. Reference lists of included studies and relevant reviews were also screened manually to identify any additional eligible studies.

Eligibility Criteria

Studies were included if they evaluated the use of POCUS for PIV line placement in trauma or emergency department patients. Eligible study designs included randomized controlled trials, observational studies, and meta-analyses published in English. Studies focusing on central venous access, surgical procedures, or non-emergency settings were excluded. Case reports, editorials, conference abstracts, and non-peer-reviewed publications were also excluded to maintain the quality and clinical relevance of the data synthesized.

Data Extraction

Data extraction was performed independently by two reviewers using a standardized form to ensure consistency and minimize bias. For each included study, relevant details were collected, including author name, year of publication, study design, population characteristics, type of intervention and comparator, outcomes assessed, key findings, and statistical data. Discrepancies between reviewers were resolved through discussion or consultation with a third reviewer when necessary. Extracted data were organized into a structured table for analysis and reporting.

Data Analysis and Synthesis

A qualitative synthesis of the findings was performed due to the heterogeneity in study designs, patient populations, operator experience, and outcome definitions across the included studies. Emphasis was placed on comparing first-attempt success rates, overall cannulation success, time to access, and procedural complications. Statistical results from meta-analyses and clinical trials were reported as odds ratios (OR), confidence intervals (CI), and p-values, where available. Trends, consistencies, and gaps in the literature were identified to highlight areas for clinical improvement and future research.

Results

Study Selection Process

**Figure 1:**
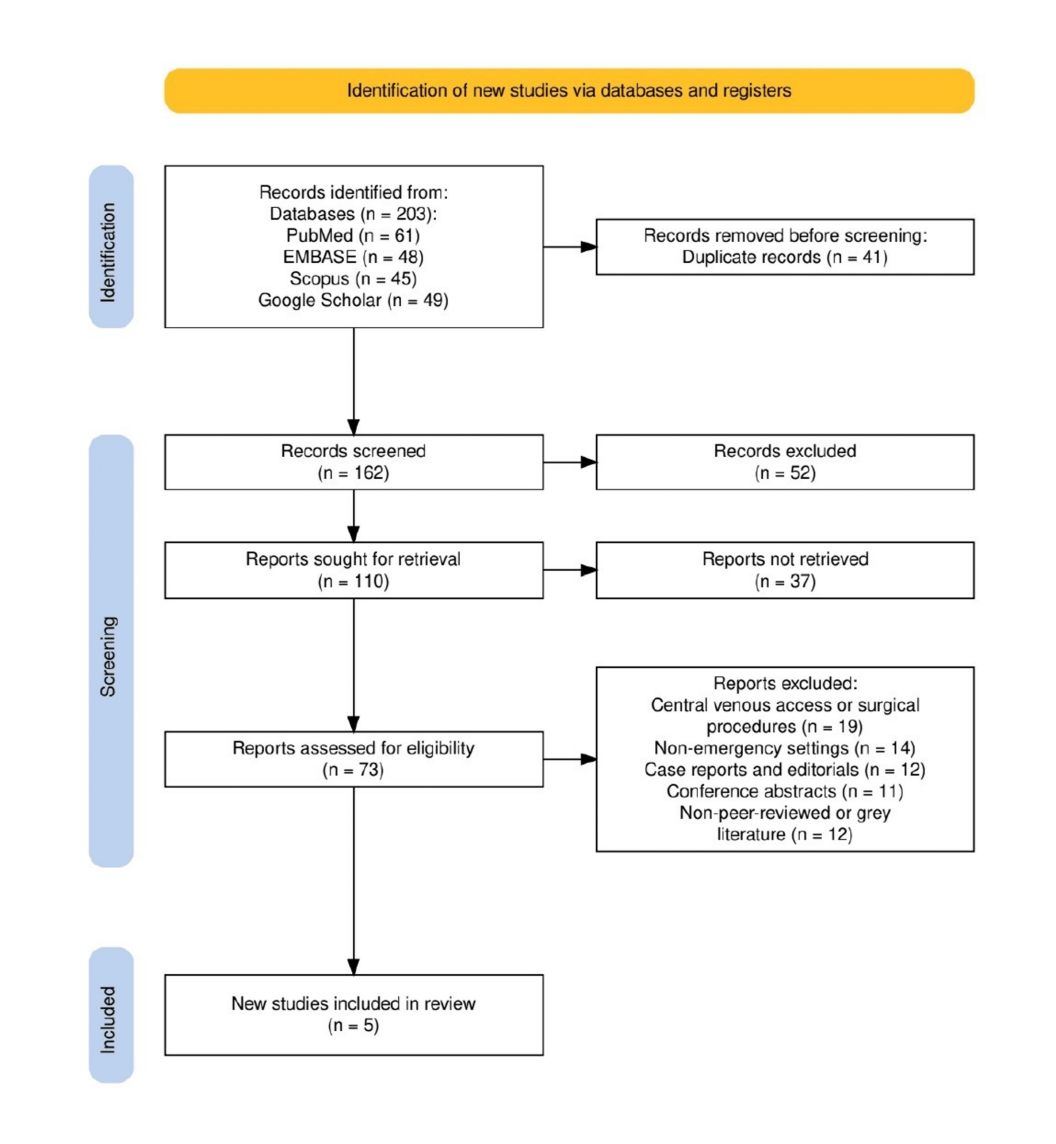
The PRISMA flowchart representing the study selection process. PRISMA: Preferred Reporting Items for Systematic Reviews and Meta-Analyses.

The study selection process followed the PRISMA 2020 guidelines and is illustrated in Figure 1. A total of 203 records were identified through database searches, including PubMed (n = 61), EMBASE (n = 48), Scopus (n = 45), and Google Scholar (n = 49). After removing 41 duplicate records, 162 unique studies were screened based on titles and abstracts. Of these, 52 were excluded for not meeting the initial inclusion criteria. Full-text reports were sought for 110 records, but 37 could not be retrieved. Among the 73 full-text articles assessed for eligibility, 68 were excluded for reasons such as focusing on central venous access or surgical procedures (n = 19), non-emergency settings (n = 14), case reports and editorials (n = 12), conference abstracts (n = 11), and non-peer-reviewed or grey literature (n = 12). Ultimately, five studies met all inclusion criteria and were included in the final review.

Characteristics of the Selected Studies

Table 1 summarizes the characteristics and findings of the five studies included in this systematic review, reflecting a diverse yet complementary body of evidence on the effectiveness of ultrasound-guided PIV access in emergency and trauma settings. The studies span various designs, including randomized controlled trials, observational studies, a quality improvement initiative, and a meta-analysis, providing a broad view of POCUS application across different clinical contexts. Sample sizes ranged from 104 to 1860 patients, with populations including adults with peripheral difficult vascular access (PDVA), pediatric emergency patients, and septic individuals. Interventions predominantly involved POCUS-guided cannulation performed by trained nurses or physicians, and outcomes assessed included first-attempt success, overall cannulation success, procedural timing, and patient satisfaction. Notably, one study reported a statistically significant reduction in time to access using bi-plane imaging (35 seconds vs. 45 seconds, p = 0.03), while another observed a high post-training success rate of 92.9% among nurses. The meta-analysis confirmed a two-fold increase in the odds of first-pass success with ultrasound guidance (OR: 2.1; 95% CI: 1.65-2.7), reinforcing the superiority of POCUS over traditional landmark techniques. However, limitations across studies included small sample sizes, lack of randomization, pediatric-only populations, and variable training protocols, which should be considered when interpreting the overall impact.

**Table 1:** The characteristics and findings of the studies included in the review. RCT: randomized controlled trial; ED: emergency department; PDVA: peripheral difficult vascular access; POCUS: pocus: point-of-care ultrasound; DIVA: difficult intravenous access; IVC: inferior vena cava; EP: emergency physician; USGPIV: ultrasound-guided peripheral intravenous; OR: odds ratio; CI: confidence interval; SMD: standardized mean difference; PIV: peripheral intravenous.

Study (First author, year)	Study design	Setting/population	Intervention (I)	Comparison (C)	Outcomes measured	Key findings	Statistical data	Limitations
Baion et al. (2023) [5]	RCT	442 adult ED patients with peripheral difficult vascular access (PDVA)	POCUS-guided bi-plane imaging	POCUS-guided mono-plane imaging	First-attempt success, time to access, complications	No significant difference in success rate; bi-plane faster	Success: 73.3% vs. 68.3% (p = 0.395); time: 35 seconds vs. 45 seconds (p = 0.03)	Single-center; limited to PDVA; short-term outcomes only
Jamal et al. (2023) [6]	Observational (pre/post)	210 pediatric ED patients (65.2% with DIVA)	Nurse-performed POCUS after training	No direct control	First-attempt success, overall success, DIVA score impact	High first-pass and overall success; effective in high DIVA	First-pass: 86.5%; overall: 91.9%; two attempts: 98.96%;, DIVA mean: 4.78	No randomization; pediatric-only; lacks a comparator group
Kalam et al. (2023) [7]	Quality improvement study	104 adult septic ED patients (mean age = 60.7)	Nurse-performed POCUS (IVC + lung)	EP clinical judgment without POCUS	Agreement with EP, fluid management changes	99.1% agreement; changed or supported treatment in most cases	EP agreement: 99.1%; management changed: 83.7%; confidence ↑: 96.6%	Not PIV-focused; small size; non-randomized
McKinley et al. (2024) [8]	Prospective observational	16 nurses; 200 total USGPIV attempts	Nurse USGPIV training + credentialing	None (training-based)	Success during and after training, time to credential	80% overall success; 92.9% after 10 successful attempts	Post-training success: 13/14 (92.9%); median attempts: 11; time: 4.13 hours	Pilot study; no control; not trauma-specific
Tran et al. (2021) [9]	Systematic review & meta-analysis	10 studies; 1860 adult ED patients (52% female)	Ultrasound-guided PIV cannulation	Standard of care (landmark technique)	First-attempt success, # of attempts, satisfaction	USGPIV doubled first-pass success; fewer attempts; better satisfaction	OR for first success: 2.1 (95% CI: 1.65–2.7); attempts: SMD -0.272 (p = 0.047); satisfaction: SMD 1.467 (p < 0.001)	Limited to studies up to 2020; variable training across studies

Quality Assessment

As summarized in Table 2, the included studies underwent quality appraisal using validated tools appropriate to their respective study designs. The randomized controlled trial was assessed using the Cochrane Risk of Bias 2.0 tool [10] and demonstrated a low risk of bias across all domains, indicating high methodological rigor. Observational studies were evaluated using the National Institutes of Health (NIH) Quality Assessment Tool, revealing moderate overall quality due to limitations such as the absence of comparator arms and potential confounding factors, despite clear reporting and well-defined outcomes. The quality improvement study, assessed with the Joanna Briggs Institute (JBI) Checklist for Quasi-Experimental Studies, was rated as moderate quality, owing to its small sample size and non-randomized design. Lastly, the systematic review and meta-analysis met most of the Assessment of Multiple Systematic Reviews 2 (AMSTAR 2) criteria, showing strong methodological transparency and low risk of bias. Overall, the quality of evidence was rated as moderate to high, supporting the reliability of the findings presented in this review.

**Table 2:** Quality appraisal of the included studies. RCT: randomized controlled trial; QI: quality improvement; PIV: peripheral intravenous; RoB 2.0: Risk of Bias 2.0 (Cochrane tool for RCTs); NIH Tool: National Institutes of Health Quality Assessment Tool; JBI: Joanna Briggs Institute; AMSTAR 2: Assessment of Multiple Systematic Reviews 2.

Study (author, year)	Study design	Tool used	Selection bias	Performance bias	Detection bias	Reporting bias	Confounding	Overall quality rating	Comments
Baion et al. (2023) [5]	RCT	Cochrane RoB 2.0	Low	Low	Low	Low	Not applicable	High	Well-conducted RCT with adequate blinding and outcome measures.
Jamal et al. (2023) [6]	Observational (pre/post)	NIH Tool	Moderate	Not applicable	Low	Low	Moderate	Moderate	No control group; pediatric-only, but clear outcome reporting.
Kalam et al. (2023) [7]	QI study	JBI checklist	Low	Low	Low	Moderate	Moderate	Moderate	Small sample size; not PIV-focused but relevant in context.
McKinley et al. (2024) [8]	Prospective observational	NIH Tool	Low	Not applicable	Low	Moderate	Moderate	Moderate	Training-focused pilot with clear results; lacks a control arm.
Tran et al. (2021) [9]	Systematic review & meta-analysis	AMSTAR 2	Low	Low	Low	Low	Low	High	Comprehensive review; robust methodology and statistics.

Discussion

This systematic review highlights the effectiveness of POCUS in enhancing PIV line placement, particularly in emergency and trauma settings. Across the included studies, POCUS was consistently associated with higher first-attempt success rates and fewer cannulation attempts compared to standard landmark-based techniques. The randomized controlled trial by Baion et al. [5] demonstrated a first-attempt success rate of 73.3% in the bi-plane POCUS group versus 68.3% in the mono-plane group, although the difference was not statistically significant (p = 0.395), while time to access was significantly faster with bi-plane imaging (p = 0.03). Jamal et al. [6] reported a remarkable 86.5% first-attempt success rate and a 98.96% success rate within two attempts following a nurse POCUS training program in a pediatric ED. The meta-analysis by Tran et al. [9] provided high-level evidence, confirming that ultrasound-guided PIV cannulation nearly doubled the odds of first-pass success compared to the standard of care (OR: 2.1; 95% CI: 1.65-2.7; p < 0.001), while also improving patient satisfaction and reducing the number of attempts. Together, these findings affirm the clinical value of POCUS for vascular access in emergency care.

Our findings are largely congruent with the broader literature, reaffirming that POCUS-guided cannulation offers superior outcomes in difficult IV access (DIVA) cases. The meta-analysis by Tran et al. [9] consolidates data from 10 studies and strongly supports POCUS over standard techniques, aligning with the success rates observed in more recent clinical trials. While studies like Kalam et al. [7] focused on fluid management rather than direct IV cannulation, their findings on nurse-performed POCUS indirectly support its procedural reliability and clinical utility in septic patients, with emergency physicians agreeing with nurse assessments in 99.1% of cases. The inclusion of pediatric patients in Jamal et al.'s [6] study adds a novel perspective, as it demonstrated the utility of POCUS in a younger population with inherently challenging vascular anatomy. Additionally, McKinley et al.'s [8] training-focused study confirms that even novice nurses can achieve high success rates (92.9% post-credentialing), supporting the adaptability of POCUS protocols across different emergency care subgroups.

The evidence from this review carries strong implications for emergency and trauma care, where time-sensitive vascular access is critical for the administration of fluids, medications, and resuscitative therapies. POCUS-guided IV access provides a faster, safer alternative to central venous access, especially in trauma patients with DIVA, potentially reducing central line-associated complications and procedural delays [11]. The consistent improvements in success rates, as seen in both adult and pediatric populations, support a shift toward integrating POCUS as a standard vascular access modality in emergency protocols. Furthermore, Baion et al.’s [5] comparison between bi-plane and mono-plane imaging introduces nuance into clinical decision-making; although both techniques were effective, bi-plane imaging marginally reduced time to access, which may hold significance in high-acuity cases. These findings advocate for broader institutional adoption of POCUS, supported by formal training programs for both physicians and non-physician providers.

This review also emphasizes the feasibility of implementing POCUS in diverse clinical settings with minimal training. McKinley et al. [8] demonstrated that a two-hour didactic and hands-on session, followed by four hours of supervised practice, was sufficient for emergency nurses to reach a median of 11 successful attempts, achieving an 80% overall success rate. Similarly, Jamal et al.’s [6] training program for pediatric ED nurses resulted in success within two attempts in nearly 99% of cases. These outcomes highlight that with structured training, POCUS can be safely and effectively used by nurses, thereby expanding the procedural capabilities of the emergency workforce. This is particularly important in resource-constrained environments where anesthesiologists or vascular access specialists may not be readily available [12]. Standardizing credentialing protocols and ensuring accessibility to ultrasound equipment can significantly optimize workflow, reduce patient wait times, and improve overall care in high-pressure trauma settings.

This systematic review benefits from the inclusion of recent, high-quality clinical trials and a meta-analysis that provide robust statistical evidence supporting the efficacy of POCUS-guided peripheral IV access. The review incorporates real-world data from emergency departments, including nurse-led interventions, enhancing its practical relevance. The use of well-defined inclusion and exclusion criteria and a clear PICO framework strengthens the methodological rigor and ensures focused analysis. However, limitations include a relatively small number of randomized controlled trials specific to trauma patients, and variability in provider skill levels and institutional protocols. Some studies combined pediatric and adult populations, potentially affecting generalizability, while others, like Kalam et al.'s study [7], focused more on fluid assessment than direct cannulation. Additionally, several observational studies lacked comparator arms, limiting their strength of inference.

Future research should prioritize large-scale, multicenter randomized controlled trials focusing specifically on trauma patients to validate these findings across diverse settings. Long-term outcomes such as catheter dwell time, complication rates, and infection risk should be assessed to determine the sustained effectiveness of POCUS-guided cannulation [13]. Evaluating the cost-effectiveness of training programs and creating standardized curricula will be essential to support widespread implementation. Moreover, studies exploring POCUS use in prehospital, disaster, or austere environments could help extend its benefits to broader emergency care contexts.

## Conclusions

This systematic review underscores the clinical utility and procedural advantages of POCUS for PIV access in emergency and trauma settings. The evidence demonstrates that POCUS significantly improves first-attempt success rates, reduces the number of cannulation attempts, and enhances procedural efficiency, particularly in patients with difficult vascular access. Moreover, the feasibility of training non-physician providers, such as nurses, to perform ultrasound-guided cannulation broadens its applicability across healthcare systems, even in resource-limited environments. Supported by both randomized trials and meta-analyses, the findings advocate for the integration of POCUS into standard emergency department protocols, potentially transforming vascular access strategies and improving patient care outcomes in time-critical scenarios.
